# Editorial for the Abstracts of the 21st Meeting of the Association of Academic European Urologists [European Urology Open Science 2024;60 (Suppl 1):S1-39]

**DOI:** 10.1016/j.euros.2024.03.002

**Published:** 2024-03-15

**Authors:** John Heesakkers, Frans Debruyne, David Castro Diaz

**Affiliations:** Association of Academic European Urologists

Dear Reader,

We herewith present you the abstracts of the 21st meeting of the Association of Academic European Urologists (AAEU) that was held in Santa Cruz de Tenerife in December 2023. The AAEU is an association of leading academic urologists from Europe, dedicated to research of all aspects of disorders of the urogenital tract. The objectives of the AAEU are to promote the role, the position and the activities of academic urology in Europe. The AAEU tries to achieve this by:

• Promoting clinical and fundamental scientific urological programmes and achievements in academic urological departments;
• Providing a forum for presentation and discussion of academic achievements in the field of urology and related areas;
• Communicating these various goals via the annual meeting, assigned journals and communication papers

Every year a two-day meeting is organised in which the members and guests present their own research in every field of urology. This research is original and new. All AAEU members and guests discuss the presented findings in a plenary setting. This implies that also those urologists that are no expert in the field of the presented topics, contribute to this discussion from their own perspective. This results in lively, high-quality discussions in which all aspects of the tackled issue are highlighted. As such, this is an integrative approach of an urological problem that gives direction for future consecutive research and clinical excellence. Therefore, the concept is well perceived by all participants.

In Santa Cruz de Tenerife, various topics were presented. The AAEU tries to balance the annual meeting with respect to type of urological topics, the topographic spread of the participants, gender and ethnic background. Currently, there are 85 active members. Looking at the final result of the scientific meeting, everyone can judge to what extent the aims were achieved.

In total we received 57 submitted abstract of which 38 abstracts were selected for presentation.

34 authors gave permission to publish their abstract in this issue of European Urology Open Science. Of the presented abstracts, 21 were about an oncological topic, 17 had a functional urological topic, 5 had a mixed topic. 12% of the presentations was presented by women.

Dear readers, with this supplement we offer insight in the dynamics of AAEU, and we hope that we may welcome you one day as our guest.

